# Pleiotropic Activities of HGF/c-Met System in Testicular Physiology: Paracrine and Endocrine Implications

**DOI:** 10.3389/fendo.2014.00038

**Published:** 2014-04-03

**Authors:** Giulia Ricci, Angela Catizone

**Affiliations:** ^1^Department of Experimental Medicine, School of Medicine, Second University of Naples, Naples, Italy; ^2^Department of Anatomy Histology, Forensic Medicine and Orthopedics, School of Medicine, “Sapienza” University of Rome, Rome, Italy

**Keywords:** HGF, c-Met receptor, testis, male gonad development, testicular cell differentiation, sex hormones

## Abstract

In the last decades, a growing body of evidence has been reported concerning the expression and functional role of hepatocyte growth factor (HGF) on different aspects of testicular physiology. This review has the aim to summarize what is currently known regarding this topic. From early embryonic development to adult age, HGF and its receptor c-Met appeared to be clearly detectable in the testis. These molecules acquire different distribution patterns and roles depending on the developmental stage or the post-natal age considered. HGF acts as a paracrine modulator of testicular functions promoting the epithelium–mesenchyme cross-talk as described even in other organs. Interestingly, it has been reported that testicular HGF acts even as an autocrine factor and that its receptor might be modulated by endocrine signals that change at puberty: HGF receptor expressed by Sertoli cells, in fact, is up-regulated by FSH administration. HGF is in turn able to modify endocrine state of the organism being able to increase testosterone secretion of both fetal and adult Leydig cells. Moreover, c-Met is expressed in mitotic and meiotic male germ cells as well as in spermatozoa. The distribution pattern of c-Met on sperm cell membrane changes in the caput and cauda epididymal sperms and HGF is able to maintain epididymal sperm motility *in vitro* suggesting a physiological role of this growth factor in the acquisition of sperm motility. Noteworthy changes in HGF concentration in seminal plasma have been reported in different andrological diseases. All together these data indicate that HGF has a role in the control of spermatogenesis and sperm quality either directly, acting on male germ cells, or indirectly acting on tubular and interstitial somatic cells of the testis.

## The Hepatocyte Growth Factor Machinery and Its Biological Functions

The hepatocyte growth factor (HGF) is a pleiotropic cytokine originally purified as a potent mitogen for hepatocytes (1, 2) and subsequently identified as a “scatter factor” (3, 4). HGF is synthesized as an inactive single chain precursor that is cleaved to acquire the bioactive disulfite-linked heterodimeric form (2, 5). One of the most recognized activators of HGF precursor is the HGF activator protein (HGFA), which is a serine protease able to cleave immature HGF precursor to form a mature bioactive HGF (6). Interestingly, HGF activation may be provided also by active metalloproteinases (MMP2 and MMP9) and by plasminogen activator (PA). More recently, it has been discovered also an inhibitor of HGF activation (called HGF inhibitor or HAI) that is a serine protease inhibitor that blocks HGFA activation (7). The modulation of HGFA and HAI in the tissue microenvironment is able to maintain the correct HGF availability since HGF has been established as an important factor for tissue homeostasis (8).

c-Met is the unique HGF receptor and it is normally expressed by cells of epithelial origin whilst HGF expression has been mainly found restricted to cells of mesenchymal origin. c-Met receptor presents tyrosine-kinase activity and, upon HGF stimulation, this receptor triggers several transduction pathways responsible for its multiple biological responses including proliferation, motility, migration, morphogenesis, tubulogenesis, differentiation, and angiogenesis (8–10). In particular, c-Met activation by its ligand HGF triggers transphosphorylation of the catalytic tyrosines Tyr 1234 and Tyr 1235, which positively modulate its enzymatic activity. c-Met c-terminal tail contains tyrosines Tyr 1349 and Tyr 1356, which represent, when phosphorylated, the multifunctional docking site of the receptor. These two amino acidic residues are able to recruit several transducers and adaptors after c-Met activation, thus explaining the whole spectrum of pleiotropic biological activities exerted by HGF/c-Met system (11). These transducers interact with the intracellular multi-substrate docking site of c-Met either directly, such as GRB2, SHC, SRC, and the p85 regulatory subunit of phosphatidylinositol-3 kinase (PI3K), or indirectly through the scaffolding protein Gab1 such as PLC-γ (12–17).

c-Met knock-out mice have provided only partial information in the understanding of the role of HGF/c-Met system in the embryonic development of mammals since this animal model showed an embryonic lethal phenotype due to severe placental defects. However, some information was drawn by these knock-out animals indicating an essential role of this growth factor in gastrulation, angiogenesis, myoblast migration, and liver development (12, 18, 19). The study of HGF/c-Met system expression during the mouse organogenesis has provided great insights in the understanding of HGF function. This system, in fact, has been found in several developing organs being HGF expressed in the mesenchyme and c-Met in the epithelial part of the developing tissue. On the basis of this preliminary observation, it has been demonstrated and well established that HGF/c-Met system mediates signal exchange between mesenchymal and epithelial cells in embryonic morphogenesis as well as in post-natal stroma–parenchyma cross-talk and tissue homeostasis (20). In addition, HGF has been demonstrated to exert unique developing capability as a morphogen of tubular structures and inducer of harmonic cell migratory activities (21–23). Even if HGF could be mainly identified as paracrine factor in the mesenchyme–epithelium cross-talk, it has been also found that this growth factor is actively delivered *via* blood vessels to injured organs allowing their repair and homeostasis (24, 25). Besides its action as a hormone, it has been demonstrated that HGF expression is regulated by blood hormones, neurotransmitters, and cytokines, such as GH (26), Norepinephrine (27), and systemic prostaglandin E (28). In addition, the discovery of HGF/c-Met system in the regulation of testis and ovary differentiation and physiology has given rise to an increasing amount of evidences that support the intriguing hypothesis of a cross-talk among gonadotropins, sex hormones, and HGF in both the female and male gonads (29, 30). This review aims to focus on what is known on the implications of HGF in the autocrine, paracrine, and endocrine regulation of testicular physiology.

## Roles of HGF in the Physiology of the Testis

Hepatocyte growth factor/c-Met system has been found expressed and active during all the phases of pre-natal and post-natal testis development. The activities of the HGF machinery on the testicular tissue vary depending on the different phases of both pre-natal and post-natal ages: these ranges from the modulation of both steroidogenesis and apoptosis to guiding mitosis, morphogenesis, and differentiation. Overall, the emerging picture suggests HGF as one of the growth factors which cooperates at different levels to support male reproductive health and is deeply involved in the harmonic control of spermatogenetic process.

### HGF/c-MET System in Testis Embryonic Development

As previously stated during pre-natal development, HGF/c-Met system is expressed and active in a wide variety of developing organs, such as the liver, lung, pancreas, intestine, and kidney. HGF transcripts were mainly found localized in the mesenchymal part of these organs whereas c-Met expression appeared mainly restricted to the epithelial portion (23), thereby indicating an important role for HGF in epithelial–mesenchymal interaction during embryonic morphogenesis. Moreover, it has been reported that HGF may induce mesenchymal to epithelial cell conversion (31). Testis develops from the collaboration and the cross-talk of intermediate mesoderm and celomic epithelium. In addition, its morphogenesis is characterized by a conversion of mesenchyme in epithelial cells (for instance Sertoli and Leydig cells) as well as by a tight cross-talk between its epithelial and mesenchymal cells. By this point of view, it may be intriguing but not surprising that HGF/c-Met system has been found during the entire period of testis embryonic development. What seems noteworthy is that the distribution patterns and functional roles of this molecular machinery change in the different morphogenetic phases of the testicular embryonic development.

#### Early testicular morphogenesis

**Figure 1:**
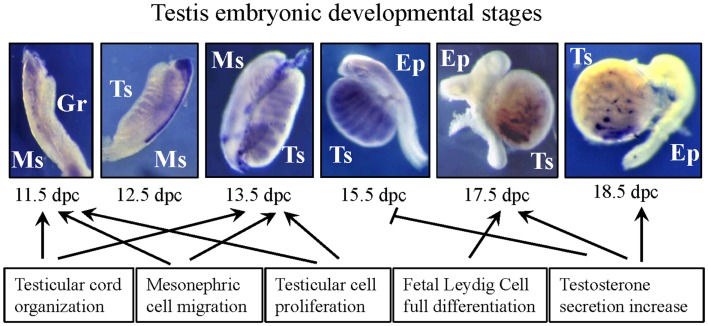
**Picture summarizing the main effects exerted by HGF during embryonic testis development**. Purple signal in the developing urogenital ridges at different pre-natal stages indicates c-Met mRNA expression. Gr, genital ridge; Ts, testis; Ms, mesonephros; Ep, epididymis.

Hepatocyte growth factor/c-Met expression has been reported starting from 11.5 dies post coitum (dpc) and continues through the entire period of pre-natal development. Actually, at 11.5 dpc only HGF is present in the celomic epithelium underlying male urogenital ridges whereas, at the same pre-natal age, c-Met is not already expressed in the gonads even if it is clearly detectable in the mesonephric mesenchyme (32, 33). One of the first specific features of male gonad development is represented by the mesonephric cell migration toward the developing testis; this cell migration begins at 11.5 dpc and was described up to 16.5 dpc in a sex-specific manner (34–37). Consistent with the observed HGF/c-Met distribution pattern in the early gonad, HGF has been established as one of the growth factors potentially involved in the chemo-attraction of mesonephric cells (32, 33, 38) thus collaborating with other growth factors such as FGF9, PDGF, and neurotrophins, to establish the male differentiation niche for somatic and germ cells (38–41) (Figure 1).

#### Testicular cord morphogenesis

Seminiferous cord formation is the critical morphogenetic event of testis development. To obtain a correct cord formation, the previously reported migration of mesenchymal cells from the mesonephros into the developing gonads is necessary (37, 42). However, this complex phenomenon requires the harmonic coordination of several biological processes such as proliferation, differentiation, and polarization of pre-Sertoli cells present in the “morphologically indifferent” gonad and their association with the primordial germ cells. The coordination of these events requires a tight epithelium–mesenchyme cross-talk that is guaranteed by the action of specific local growth factors (43–45) and HGF has been indicated as one of them. Testicular cords begin to organize at 12.5 dpc. At the same developmental age, c-Met starts to be expressed by the developing testis and seems to be confined in the testicular cords of this organ. As reported for other organs, at the same stage of development, HGF expression is present in the differentiating stroma and in differentiating myoid cells confirming the capability of HGF/c-Met system to mediate epithelium–mesenchyme exchange (32, 33). Using organ culture of indifferent male urogenital ridges, it has been demonstrated that HGF is able to mediate testis differentiation and testicular cord formation in *ex vivo* organ culture condition (32, 33, 38). Since this *ex vivo* differentiation is the result of the coordination of cell migration, cell proliferation, and tubulogenesis, using *in vitro* assays able to discriminate between these different phenomena, it has been established that HGF is able to trigger all these events, again confirming the multiple biological activities that are mediated by this molecular machinery (32, 33, 38) (Figure 1).

#### Fetal Leydig cell differentiation and endocrine implications

c-Met receptor continues to be expressed by testicular cord up to 15.5 dpc. It is interesting to notice that in the late part of pre-natal development (since 17.5 dpc to birth) c-Met distribution pattern drastically changes, being down-regulated in the testicular cords and up-regulated in the interstitial fetal Leydig cells (46). HGF is always present in the interstitial compartment, but, interestingly, it is not produced by mouse fetal Leydig cells. Thus HGF acts as a paracrine factor even on differentiating fetal Leydig cells reproducing in the interstitial compartment the epithelium–mesenchyme cross-talk present between interstitium and testicular cords in the embryonic stages before this pre-natal age. Actually, steroid-producing fetal Leydig cell lineage starts to be detectable in the testis early on 12.5 dpc but their differentiation process involves all the further stages of pre-natal development when they increase in number and gradually acquire the capability to modulate androgen secretion in response to local and endocrine cues (39, 47, 48). It is worth to highlight that when mesenchyme derived fetal Leydig cells start to acquire their fully differentiated phenotype in late embryogenesis (49), they start to express c-Met on their surface. The latter observation allowed to hypothesize a role for this growth factor in the modulation of testicular pre-natal endocrine function. In rodents, in fact, fetal masculinization and increase of plasma androgens occur before the hypothalamic–pituitary–gonadal axis to be functional (50) since LURKO mice (that lack the luteinizing hormone receptor) have normal androgen levels and testicular phenotype at birth (51). Thus there is a common agreement in the scientific community that states the differentiation of fetal Leydig cells is under the control of local growth factors and HGF seems to be one of them. In fact, in 17.5 and 18.5 dpc testicular organ culture, HGF is able to stimulate testosterone production (46) and fetal Leydig cell survival and full development (52). Noteworthy at 15.5 dpc, when c-Met is not detectable on fetal Leydig cells but is still expressed in seminiferous cords, HGF has been demonstrated not able to modify testosterone secretion in testicular organ culture. From these data HGF can be numbered besides PDGF, DHH, TGF-β, and IGF-I as one of the growth factors responsible of the normal pre-natal steroidogenesis (39, 48, 52–55).

This phenomenon may be relevant for the onset of the first cross-talk among gonad, hypothalamus, and pituitary gland, and potentially involved in the acquisition of secondary sex-specific features and brain testosterone imprinting (Figure 1).

### HGF/c-MET system in the physiology of post-natal testis

Even if the studies on pre-natal morphogenetic functions of HGF have been established using mouse models, the post-natal roles of HGF on testicular physiology have been determined mainly using rats since testicular cell isolation is better characterized for this animal model. Intriguingly, many parallel mechanisms exist comparing ovarian and testicular function and some of them, with particular attention to endocrine function, will be highlighted in this part of the review.

#### Sertoli cells

Correct Sertoli cell differentiation is crucial in order to maintain the local microenvironment necessary to sustain spermatogenetic process (56, 57). Several growth factors have been described as local modulators of Sertoli cell physiological niche and HGF can be numbered in this cohort since it has been demonstrated as a paracrine and autocrine modulator of Sertoli cell physiology. In the rat, c-Met mRNA and protein were detected in the post-natal testis starting from 10 dies post partum (dpp) even if they appear on Sertoli cells not before 25 dpp and their expression increases at 35 dpp. As previously reported in the embryonic testis, also in the post-natal testis the main source of HGF seems to be represented by the interstitial cells and by the myoid cells, reproducing the epithelium–mesenchyme cross-talk observable during the morphogenesis of the testis. Despite this observation, in the post-natal testis, HGF has often been demonstrated to be expressed also by the same cell lineages that express c-Met and Sertoli cells are not an exception. These data suggest that HGF levels could be finely regulated by different testicular cell types and that the action of this factor is not only paracrine but seems to be also autocrine and maybe endocrine. Interestingly, dissociated rat Sertoli cells as well as the Sertoli cell line SF7 cultured in the presence of HGF, are able to organize in tubular-like structures showing, even in the post-natal testis, the morphogenetic and tubulogenic ability of this growth factor (58, 59). This observation demonstrates that HGF is able to guarantee the right cue for the maintenance of correct Sertoli cell polarization that is one of the parameters showing full differentiated Sertoli cell phenotype. These data together with the age of onset of Sertoli cell sensitivity to HGF have strongly suggested that this growth factor may be involved in testicular cord lumen and blood–testis barrier (BTB) formation. This particular topic will be fully expanded in a paragraph below.

**Figure 2:**
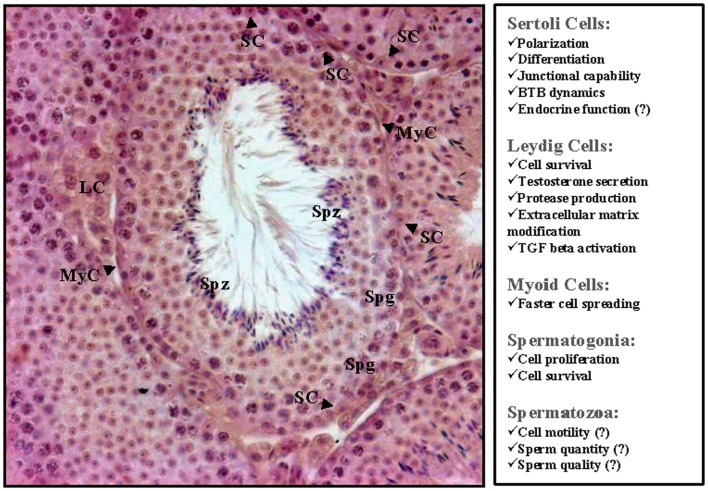
**Picture summarizing the main effects exerted by HGF on post-natal testis derived cells**. A hematoxylin–eosin stained section of rodent adult testis is also reported. SC, Sertoli cells; LC, Leydig cells; MyC, myoid cells; Spg, spermatogonia; Spz, spermatozoa.

Sertoli cells are one of the key somatic actors of endocrine hypothalamus–pituitary–gonadal axis cross-talk being able to produce and secrete estrogens in response to FSH and thus to regulate the spermatogenetic process. As HGF has been proposed as one of the factors able to modify Sertoli cell physiology and to maintain their differentiated phenotype, it is conceivable to hypothesize that this factor has a role in the modulation of gonad–pituitary cross-talk. It was suggested by Zachow and Uzumcu in 2007 (30), that there is an intriguing parallelism between male and female gonad that hypothesizes some endocrine implications of HGF in the physiology of both organs. It has been reported, also, that HGF can down-modulate ovarian steroidogenesis suppressing FSH-dependent 17β-estradiol production by directly impairing CYP19 enzyme (60, 61). It is well known that Sertoli cells represent the testicular counterpart of granulosa cells and are the testicular source of 17β-estradiol. Sertoli cells are capable to produce the greatest quantity of 17β-estradiol in the first 10–20 days of post-natal age (62). Interestingly, HGF together with c-Met is not present on Sertoli cells at post-natal day 10 (63, 64) whilst both the receptor and the ligand appear expressed by Sertoli cells since post-natal day 25 (64, 65), which is the time when 17β-estradiol production by Sertoli cells begins to be reduced (62). All together these observations allow to hypothesize that HGF could locally control *in vivo* Sertoli cell 17β-estradiol production in a similar way of what observed in granulosa cells. Consistent with these data, FSH has been demonstrated able to up-regulate c-Met expression in Sertoli cell cultures (65) whose activation in turn suppresses, following this hypothesis, Sertoli cells 17β-estradiol synthesis. The proposed mechanism, that deserves further investigations, suggests that HGF/c-Met system may be responsible for a local modulation of FSH-dependent estrogen production, modulating both enzyme activity and c-Met receptor availability (Figure 2).

#### Germ cells and spermatozoa

It has been reported by several groups that c-Met is expressed in both human and rodent male germ cells: this observation is interesting since it means the expression of this receptor is conserved at least among mammals (66–68). In rats, this receptor is always present during spermatogenetic process from spermatogonia to spermatozoa. HGF has been shown to control germ cell mitotic activity being able to significantly increase spermatogonial cell proliferation from 8 to 30 days old rat testis in *ex vivo* organ culture. This result on spermatogonia cells appears to be more relevant highlighting that the HGF activator inhibitor (HAI-2) is expressed exclusively in primary spermatocytes (69) strongly indicating that mitotic germ cells need HGF signal whereas at the beginning of meiotic process this proliferating cue needs to be inhibited in order to allow germ cell meiotic entry.

Germ cell apoptosis is finely controlled in the testis in order to guarantee the best selection of differentiating male gametes and HGF has been demonstrated to be involved even in the control of this biological process. This growth factor, in fact, has been demonstrated to act also as a survival factor for male germ cells since it is capable to significantly decrease germ cell apoptosis (67). The lowest protective effect (35% reduction of apoptosis) was found in late prepubertal rats and this finding is probably due to the fact that the highest level of apoptosis has been described at this age (70). Alternatively, this observation could be due to the presence of a more complex network of pro-apoptotic and anti-apoptotic factors present in the older animals (Figure 2). This result on healthy animals is reinforced by Goda and coworkers who studied a rat experimental cryptorchid model (71). This pathology is characterized by spermatogenic failure and germ cell loss. The adenovirus-mediated HGF gene transfer in the testis of these animals induced over-expression of HGF and significantly decreased apoptotic germ cell number, restoring spermatogenesis and testicular weight.

It is well known that germ cell proliferation and survival are controlled by endocrine signals: for instance FSH stimulates spermatogonial proliferation both *in vivo* and *in vitro* (72–74). In addition, testosterone and FSH regulate germ cell apoptosis (75, 76) probably acting via somatic cells considering the absence on the germ cells of their respective receptors (77, 78). By this point of view, HGF may be considered one of the local cytokines already identified (57, 79–83) that collaborates with the endocrine signals to promote the correct male germ cell homeostasis.

There has not been a report of any effect of HGF on meiotic germ cells, even if they express c-Met receptor on their surface (67). However, several groups have reported that c-Met is expressed on the surface of rat (66), and human epididymal spermatozoa (68, 84) and that HGF is present in mouse (85), rat (66), and human (86) epididymis. It is fair to say that literature data on the role of HGF on epididymal spermatozoa are often controversial. The first finding that strongly suggested a role of HGF in epididymal sperm maturation was reported by Naz and coworkers in 1994 (85). This group showed a specific region distribution pattern of HGF in the mouse epididymal tract with the highest levels of the growth factor in the distal corpus and cauda, where sperms acquire their motility. In the same study, HGF was found able to slightly induce cell motility on immotile sperms. Noteworthy, also c-Met distribution pattern on sperm surface seems to change from testicular to caput and cauda epididymal spermatozoa in rats (66). In addition, the previously mentioned effect on sperm motility was, at least in part, confirmed on rat epididymal sperms in which HGF has a positive effect on the *in vitro* maintenance of epididymal sperm motility even if, actually, the factor was not able to significantly increase the percentage of motile cells (66). Interestingly, c-Met receptor on human sperm has been found activated indicating that the HGF/c-Met system is functionally active in epididymal spermatozoa (84). However, the same motogenic effect reported in rodents failed to be demonstrated on human sperms (87) and the role of this growth factor on human sperm physiology is still a matter of debate (Figure 2). It was suggested that modifications in HGF seminal plasma concentration could be related with different andrological diseases and male infertility, but actually as in this case conflicting data are reported in the literature (87–89). It is fair to notice that male infertility could be due to really different causes: it is likely that the reported literature controversy may depend on the difficulties in classification of andrological diseases considered eligible for this particular study by the different research groups. Further studies are needed to better clarify this point that deserves to be deeper investigated.

#### Blood–testis barrier

The described morphogenic and motogenic effect on cultured pre-natal and post-natal Sertoli cells allowed to hypothesize that HGF may modulate junctional capability of this cell type. It is well known, in fact, that HGF is a “Scatter Factor” able to modulate junctional behavior of target cells. In adult mammalian testes, Sertoli cells form junctional complexes with neighboring Sertoli cells that have been described from a long time (90). These junctional complexes consist of tight junctions and testis specific cell to cell actin based anchoring junctions which are both involved in the formation of the BTB. BTB separates the seminiferous epithelium in two different niches: the basal compartment, that encloses mitotic spermatogonia, and the adluminal compartment that encloses male meiotic germ cells. BTB integrity is necessary to allow the correct spermatogenic process, but its structure is highly dynamic. Junctional complexes of BTB, in fact, are able to disassemble and reassemble to allow the passage of pre-leptotene spermatocytes across the barrier (90, 91). In the last years, multiple reports from different laboratories have indicated that BTB permeability and physiological dynamics are regulated by a complex interaction of bioactive molecules including gonadotropins, testosterone, TGF-β, TNF-α, and interleukin-1a (92–98). Since 2008, HGF must be included in this number of factors (58, 99). In pubertal and adult rats it has been reported, in fact, that HGF is involved in the disassembling of the polygonal structures formed by occludin around the Sertoli cells. Moreover, it was demonstrated a quantitative occludin decrease in the tubules cultured in the presence of HGF by means of both confocal microscopy and Western blot analysis. In addition, HGF modifies the position of the tight junctions: it is indicated by the shift in the position of the occludin within the tubule treated with the growth factor compared to the controls. These data indicate a role of HGF in the modification of Sertoli cells junctional behavior. Interestingly, in adult rats HGF is maximally expressed at stages VII–VIII of the cycle, when germ cells traverse the BTB, whereas its levels fall in the subsequent stages IX–XII and XIII–I (58). This observation gives rise to the intriguing hypothesis that HGF produced by Sertoli cells could autocrinally regulate BTB in a stage dependent manner. It is relevant to highlight that HGF mediated BTB dynamism may be potentially due not only to a direct motogenic effect of HGF on Sertoli cells but also to the capability of HGF to modify the seminiferous tubule microenvironment promoting the increase of TGF-β active fraction. This phenomenon could be ascribed, at least in part, to the uPA level increase mediated by HGF in seminiferous tubule cultures (58, 99) (Figure 2).

#### Myoid cells

Myoid cell lineage was the first isolated testicular cell type in which HGF/c-Met system has been discovered (63). Even if HGF has always been found expressed both in pre-natal and post-natal myoid cells (33, 63, 64), c-Met was detectable only in post-natal cells indicating a paracrine function during embryonic development and a paracrine/autocrine role in post-natal testis. Noteworthy, myoid cells isolated from prepubertal rat testis (10 and 20 days old rats) express c-Met receptor at high level whereas the expression level decreases in pubertal and adult myoid cells (35 and 60 days old rats). Consistent with these results, HGF is able to induce a faster cell spreading of prepubertal myoid cells but not able to modify this parameter on myoid cells isolated from pubertal animals (Figure 2) (64). Despite myoid cells were the first testicular lineage in which HGF/c-Met system has been described, the role exerted by this machinery in their physiology needs to be better investigated. Probably, one of the roles exerted by myoid cells is to provide a source of this factor both for Leydig and Sertoli cells.

#### Leydig cells

As well as mouse fetal Leydig cells, also Leydig cells isolated from pubertal rats expressed c-Met receptor (100). Physiological activity of HGF on pubertal rat Leydig cells presents some similarities with their fetal counterparts: in particular as HGF has been demonstrated to promote basal testosterone secretion and Leydig cell survival (100) both in Leydig cell primary culture and in *ex vivo* organ culture. Intriguingly, the steroid modulator activity is not confined in male gonad but was also described in theca cells, which are the Leydig cell ovarian counterparts. In rat theca cells, in fact, HGF suppressed LH-dependent androsterone secretion, while stimulated basal and LH-induced progesterone production (60). It is fair to highlight the parallelism between male and female gonad and to notice that steroid production modulation in response to local cues is quite relevant for gonad physiology since sex hormones are not only important for endocrine homeostasis via pituitary–gonadal axis cross-talk but also act as paracrine regulators of male and female gametogenesis. In addition, local increase of testosterone could be important for BTB dynamism that, as previously stated, was also directly modulated by HGF.

Besides its effect on testosterone secretion, it is worth mentioning that HGF exerts a broader effect on Leydig cell secretory activities: HGF administration to isolated rat Leydig cells was able to increase the secretion of active form of several proteases such as MMP2 and uPA (101). Interestingly, HGF administration was also able to increase active TGF-β. Viewed together these data suggest that increased amount of active TGF-β could not be a direct effect of HGF but a consequence of the HGF-dependent increased uPA and MMP2 activity (102–104).

Different types of molecules can be substrates of MMPs including growth factors, tyrosine-kinase receptors, extracellular matrix proteins, and cell adhesion molecules. HGF administration on isolated Leydig cells significantly reduces the amount of fibronectin indicating that this growth factor might modify interstitial extracellular matrix components and in turn changes significantly adhesive microenvironment and cytokine availability (Figure 2). It is fair to highlight that extracellular matrix homeostasis is necessary for spermatogenesis and that a thickened lamina propria has been reported associated with impaired spermatogenesis (105) and that HGF has demonstrated to have anti-fibrotic effect in the regulation of extracellular matrix composition even in other organs (8). All together, the presented data indicate a relevant role of HGF in the regulation of Leydig cell metabolic activities and in the composition of the interstitial tissue. The reported results strongly indicate that HGF/c-Met system is implicated in the local control of endocrine testicular machinery.

## Concluding Remarks

Hepatocyte growth factor has been well established as a key regulator of the development and homeostasis of many organs. An increasing amount of evidences is demonstrating its important role in several aspects of pre-natal and post-natal testicular physiology. A huge job has been done but we must deal with a major one to figure out what might be the implications of this factor in the reproductive health of human beings. A better understanding of the molecular mechanisms carried out *in vivo* by this growth factor could be a useful prerequisite in order to address idiopathic andrological diseases.

## Conflict of Interest Statement

The authors declare that the research was conducted in the absence of any commercial or financial relationships that could be construed as a potential conflict of interest. The Guest Associate Editor Gilda Cobellis declares that, despite being affiliated to the same department as author Giulia Ricci and having a collaboration in another journal, the review process was handled objectively and no conflict of interest exists.
